# Post traumatic stress disorder in postpartum: when guilt delays recovery. A case report

**DOI:** 10.1192/j.eurpsy.2025.1949

**Published:** 2025-08-26

**Authors:** R. De Hita Santillana, M. L. Costa Ferreira da Silva, M. del Valle Martin, E. Fiori

**Affiliations:** 1 Hospital Universitario Jose Germain; 2 Hospital Universitario Severo Ochoa, Leganés; 3 Universidad Europea de Madrid, Madrid, Spain

## Abstract

**Introduction:**

PTSD is a mental disorder that may develop after exposure to threatening or horrifying events. PTSD is characterized by the following: 1) re-experiencing the traumatic event or events in the present in the form of vivid intrusive memories, flashbacks, or nightmares. Re-experiencing may occur via one or multiple sensory modalities and is typically accompanied by strong or overwhelming emotions, particularly fear or horror, and strong physical sensations; 2) avoidance of thoughts and memories of the event or events, activities, situations, or people reminiscent of the event(s); and 3) persistent perceptions of heightened current threat. The symptoms persist for at least several weeks and cause significant impairment in personal, family, social, educational, occupational, or other important areas of functioning.

Some reviewes found that PTSD is prevalent during pregnancy and after birth and may increase during postpartum if not identified and treated. It can affect women, their relationship and birth outcomes aswell as infant emotion regulation and development. The findings indicate that there are links between psychological, traumatic and birth-related risk factors as well as the perceived social support and the possible PTSD following childbirth in mothers and partners.

**Objectives:**

A case report is presented alongside a review of the relevant literature regarding the prevention, diagnosis, and treatment of PTSD.

**Methods:**

We present a case report of a 34-year-old woman with no previous contact with Mental Health Services. She got pregnant unexpectedly in the context of a long stable relationship. At the beginning she was feeling uncertain about carrying on with the pregnancy but finally decided to keep it. She states she felt well throughout the pregnancy. The night after giving birth her baby suffered from a cardiorespiratory arrest, which was noticed by the nursing team but not by the mother. The baby recovered with no medical sequelae but the patient started feeling depressed and presenting anergy, apathy, irritability, flashbacks, and intrusive memories of her sick baby and insomnia, checking every hour during the night that her child was still breathing. Later she developed separation anxiety from her baby, not being able to leave her in the kindergartner nor to leave her alone with other family members (including the father). Guilty feelings were persistantly observed during the therapy sessions.

**Results:**

Psychotherapeutic and pharmacological treatment was started with moderate improvement. Since breastfeeding was a rewarding experience and enforced the mother-daughter bond it was taken in consideration for the therapeutic plan.

**Conclusions:**

The postpartum period is of special vulnerability and early treatment of symptoms in mothers is of great importance. Early diagnosis in maternity services should be a priority.

**Disclosure of Interest:**

None Declared

